# The Relation of Science to Progress in Dental Practice

**Published:** 1899-02

**Authors:** S. B. Palmer

**Affiliations:** Syracuse, N.Y.


### THE RELATION OF SCIENCE TO PROGRESS IN DENTAL PRACTICE.[1]

BY S. B. PALMER, M.D.S., SYRACUSE, N.Y.

The first dental periodical in this or any other country was issued on the 1st of June, 1839, under the title of "The American Journal of Dental Science." From that time until the present science has been a prefix used, in connection with dental literature and in discussions, to denote the highest attainments in knowledge and skill. Progress in medicine and dentistry has been slow, but once it becomes established upon a scientific basis, it remains as unchangeable as literature recorded in a dead language.

One of the advantages that science offers to dental practice is the knowledge of laws and principles which may be taught students at the outset, as unerring guides in selecting materials and remedies to meet specific conditions. One law relating to dentistry is this: "Under the same conditions, with the same materials, the same causes produce the same effects." Most of the controversy and confusion that retards progress is caused by misplacing conditions which will always give different effects.

I will not attempt to teach you operative dentistry beyond its aid in demonstrating science, as gained in my own practice. So far as this conforms to your clinical experience it may be pronounced scientific. Medical science had been victorious on many a battle-field before dentistry claimed to be a profession. Evolution in the practice of dentistry has likewise provoked opposition.

In 1874 I read a paper before the Dental Society of the State of New York. From the discussion I quote the following from a

---

[1] Read before the Northeastern Dental Association, at Hartford, October 19, 1898.

member: "Dr. Palmer has given the society his views upon the subject. He has made statements, but they all seem to me to be *ipse dixit*." That was twenty-four years ago, and the quotation there voiced the sentiment of the profession. Evolution in practice has absorbed not only those statements, but many more radical, until of late we have expressed opposition condensed into tables of physical facts that are arrayed against the so-called "electrical theory," which in no respect relate to the conditions under discussion.

Twenty-one years ago it was my mission, or perhaps misfortune, to set forth a doctrine which came to be known as "the electro-chemical" theory of caries, or secondary decay around gold fillings in the mouths of young patients. Very naturally, the theory met with earnest opposition. Gold was king of filling-materials. It represented respectable and remunerative practice. The new doctrine, from a scientific stand-point, taught that for this class of operations gold had faults. Also, for specific conditions, that amalgam was better adapted for arresting decay than gold. That was sufficient. Science had no voice in discussion. The theory was charged with debasing gold, exalting amalgam, and degrading the profession. As years went on, dental progress on various lines of investigation was under the leading of authority, conducted by able minds, with means and opportunity to do much for the profession.

It seems very strange that during so long a period any theory bearing upon a subject so important as tooth-preservation should have been disposed of by the sweeping declaration that "the theory has no scientific foundation." In charity I make allowance for prejudices which have come down through the feelings engendered during the early discussion of the "New Departure." Dr. Flagg, whom you all know, has a way of pushing things peculiar to himself, which is not always agreeable to others. In his introduction of the basal principles of the "New Departure," he said, "I am the mouth-piece of Dr. Palmer," referring to the scientific correspondence previously obtained for that occasion. His part was tabulated experience. Whatever criticisms may have been made for the vigorous work he did on that line, I believe no other man can favorably compare with Dr. Flagg in connecting science with practice. Such was the opposition to my findings then, that, if I had been as timid as a quotation to follow would indicate, I could not have been induced to be present to-night. Dr. Flagg did not profess to teach science, therefore the task has fallen upon me. And with this encouragement, opposition seems to have dwindled to tables of facts, which do not relate to the conditions.

I will in no way attempt to controvert the tables mentioned. As understood, the labor has been done in the laboratory, and the findings carefully noted. The conclusions are based, as I understand, upon experiments made to find all the properties and variations of tooth-structure under all conditions. Such investigations have been conducted with the most accurate instruments that could be made for the purpose. We are surprised at the findings that there is such a sameness. This does not in any degree cover the conditions or conflict with the findings upon vital organic matter. I worked three years in laboratory work to find that nothing could be gained from nature short of observation in the laboratory where the work was being done. The results will be given farther on. The line of division between opposition and our field of operation is between the laboratory and the chair; our observations are upon the conditions of development of teeth, and the durability of fillings in relation to various conditions. Comparatively speaking, we might as reasonably attempt to change public opinion regarding an individual's character for veracity by a post-mortem examination of the brain as to decide the proper filling-material for the various conditions of teeth by experiments in the laboratory.

As may be seen in the June issue of the *Dental Cosmos*, 1898, Dr. Black reviewed the papers containing a review of his conclusions previously published in the *Dental Cosmos*. These papers were read before The New York Institute of Stomatology, October 5, 1897, and appear in the INTERNATIONAL DENTAL JOURNAL, December, 1897. Dr. Black says, "The gentlemen writing the reviews are representative men, well known to the dental profession, and are persons whose views command great respect. They are Charles S. Tomes, of London; Dr. R. R. Andrews, of Boston, Mass.; Dr. S. B. Palmer, of Syracuse, N. Y.; Dr. James Truman, of Philadelphia, Pa.; Dr. George A. Maxfield, of Holyoke, Mass.; Dr. E. A. Bogue, of New York; and Dr. B. Holly Smith, of Baltimore, Md. While their papers are deliberate and very respectful in their tone, most of them present a strong inclination to cling to the older views, and yet a disposition to yield to the testimony adduced."

For fear of doing injustice in abbreviating the quotations presented from my paper, I give them entire, with the criticism, being confident that I could not present the distinction between Dr. Black's conclusions and my own any better: "Dr. Palmer's paper is a very curious piece of writing. He says, on page 791, 'These are facts recorded in the tables emanating from physical investigations, so carefully prepared and systematically arranged that they are under the seal of science and challenge refutation.' That was complimentary to his laboratory work. And upon the same page he also says, 'So far as the difference exists relating to the teeth which we represent, science from the physical plane is misapplied,' and says that 'the investigations which we are called upon to refute are of the earth, earthy.' And again, on page 790, 'We will now take our stand upon the animal plane where man lives and moves and has his being, which is quite above the plane upon which the conclusions under discussion were based.'

"From this it will be seen that while Dr. Palmer acknowledges the correctness of the findings as being upon what he calls the physical plane, he regards them of no consequence because he works upon the animal plane, which is higher. Still he objects to the conclusion that there is no basis for the selection and adaptation of filling-material to soft teeth, hard teeth, frail teeth, or poorly calcified teeth, for the reason that all are found to be sufficiently hard, and says, 'We know that teeth undergo changes from the time of their first calcareous deposits until the pulp, from natural limitation or from caries, or from accident, fails to perform its functions. We know that teeth at twelve years of age have not reached the limit of pulp nourishment.' An examination of the tables I have presented proves that there is a constant increase in the proportion of lime-salts from youth to old age, and Dr. Palmer ought not to represent, as he does in these sentences, that I have denied pulp-nourishment under any normal condition of the teeth, much less assert that it ceases after the age of twelve years. Proceeding directly from the last quotation Dr. Palmer says, 'We know that young teeth are softer than the same would be at maturity; also that some are free from caries, and others are badly decayed at twelve or fourteen years. We know that it is under a natural law that the latter decay when filled with gold without some insulating lining, for the following reasons: The fact that decay has attacked several teeth is an evidence that their structure is faulty. We know that dentine is a conductor when in this condition.' Dr. Palmer thus prepares the ground for the presentation of the electric theory of caries and of the selection and adaptation of filling-materials to soft teeth and to hard teeth, or to teeth not well calcified. The tables which I have presented seem not to have given enough variation in lime-salts to work his electric currents properly. He therefore escapes from the physical plane which is 'of the earth, earthy,' and rises to a higher plane, and proceeds without disturbance from the facts found to exist in the plane below. The doctor's papers are always interesting, even though we cannot all agree with his findings."

The above review of my paper shows conclusively that Dr. Black clearly understands that my conclusions are based upon experiences gained by clinically observing the effects of various filling-materials in the mouth, extending through years in the same mouth, as others have done. Facts have been thus gained which no laboratory facts can refute. And yet Dr. Black only voices all opponents for a score of years past in saying that there is no basis for the selection and adaptation of filling-materials, etc. I will add this quotation. "With our present knowledge the only basis for the selection and adaptation of filling-materials to classes of cases is the individual operator's judgment as to which he can so manipulate as to make the most perfect filling considering the circumstances, his own skill, and durability of materials." Can it be possible that this society of dentists can believe that there is no science in filling teeth? That with all the study and earnest work that has been done there are no rules, laws, or guides to offer a young graduate that would save him much chagrin and loss of patients, while waiting to obtain experimental knowledge? Can the profession believe that all must be trusted "to the individual operator's judgment?" Not only is science denied on these points, but tables are set forth to prove, as others have said, that there is no scientific foundation for theories which do not agree with misapplied facts.

I will briefly outline my belief in relation to the difference between oral electricity and electricity as understood in physics. I am well aware that this writing has no authority in our text-books, but most new discoveries have met with opposition until established in fact. By wearing plates—silver, gold, vulcanite, and aluminum—I discovered that taste of food, drinks, etc., was a sensation caused by electricity which was generated in the mouth by the bringing in contact of positive and negative elements of food, or liquids in connection with saliva.

That such currents are formed by mixing any compounds in which there is chemical change in the laboratory is well known to any one conversant with electricity. Thus the experiments I had carefully conducted both in and out of the mouth with food and saliva gave no new principles. The galvanometer was abandoned for that class of experiments. Still I believed that the electricity generated from organic elements was also organized electricity, and gave taste. The wearing of silver for two years gave some ideas, but they were not distinct. The change to a gold plate established my belief,—*i.e.*, I had the experience of a positive and a negative plate, each worn separately. Silver was positive, gold negative, which gave both poles of a battery, compared with food for the opposite pole. Silver being positive as compared with most kinds of food gave the best evidence that vital electricity was converted into physical electricity as soon as it passed through a metallic conductor or came in contact with metals. To illustrate this point I will compare it with a jet of blood flowing from an artery: it is a current of life; gather it in a vessel, and it is but a devitalized coagulated chemical compound. When a current of *vital* electricity is caused to flow through a metallic current, it is delivered as physical electricity. Without the wearing of a silver plate I should not have had any means of obtaining this knowledge. Let us attempt to make this plain by a lesson which can be appreciated by any one not wearing metal plates. First, food is both positive and negative, and saliva is an unstable fluid easily changed by contact with either positive or negative elements of food as previously mentioned. The chemical action thus set up, having no conducting poles or polarity, remains in the mass until by deglutition it passes into the stomach, its only manifestation in the mouth being taste. Many also know that a metal, when immersed in such a mass, either in the laboratory or in the mouth, would at once become charged with electricity. Should a single plate be inserted, there would be no established current. Still it would effect the compound, should the plate be either positive or negative in this respect. Let the plate be silver, there would be more electro-chemical action than if it were gold, because the silver would be positive to more kinds and conditions of food than would be the case with gold. Thus far, I trust, many of you fully understand. To continue, I will introduce an idea not found in dental text-books: That organic food generates vital or organic electricity; also that in preparation of food we indulge in adding carbon to that naturally belonging to it, as well for the energy it affords as stimulant, by increasing the current, as for delicacy of taste. Nature knows where to draw the line between organic and inorganic food. This knowledge greatly aided in the experiments in which I was engaged. Long before science was taken into account cooks toasted bread, broiled meats, and roasted coffee. This process adds carbon without disorganizing the food. The brown portions are organized carbon the same as that which is not browned. The increase of the electric current is manifested by delicacy of taste; the elements which are employed are the carbon negative, the usually cooked portions positive, with saliva for the fluid. Take for illustration one element, coffee. Imagine a cup of coffee made from the unbrowned berry, or, on the other extreme, from coffee burned in roasting; the latter would be mineral carbon. And the same principle runs through the whole series.

Carbon is not the only element suggested by nature to increase the current of vital electricity. Fruit acids, etc., chloride of sodium as seasoning of food, salting of almonds, are tolerated by nature. The galvanometer gave correct readings of all the above-named elements with saliva in the laboratory, but there was no connection with life and vitality until observations were taken from plates worn as described. Sulphur, an element little used in cooking except as an ingredient in eggs, was very important in establishing belief in vital electricity. It is known that silver is sulphuretted by sulphur; the silver plate was a galvanometer which recorded not only the increased current by taste, but electricity proper by the sensation of a mild shock, or rather a flash-like feeling unmistakable of physical electricity. Breathing while passing a sulphur spring, which was several rods from the road-side, was more distinct by taste than by smell. While silver as a whole was more or less annoying in giving metallic tastes, there were other conditions not in the least disagreeable. Nature drew the line as distinctly on that principle as she does on roasting coffee. At this point I will suggest an experiment with coffee, and not a disagreeable one at that. When enjoying a cup of black coffee without cream, sip it slowly from a spoon, and notice the thinning of the saliva and the taste immediately following; add cıeam, and it will be found a partial insulator.

For the same delicacy of taste sugar is burned in brandy; more carbon is added. For this purpose pewter beer-mugs were once so popular; the ale was truly electrified, being charged in the mug and discharged in the mouth, as demonstrated in taste. Thus far nature admitted electricity. Should the voltage be increased, taste would be abnormal and the drink or food would be rejected.

This allows us to bring out a lesson in regard to electric currents in the body. All remember that dentistry gave to the State of New York electrocution. The late Dr. A. P. Southwick knew that electricity from a dynamo would kill quickly. Since then another discovery has been introduced, one of its virtues being to electrocute pulps in teeth by cataphoresis. It is also used in milder form to destroy sensibility in dentine for painless excavating. I am not discussing cataphoresis. It is well to remember that electricity kills the body, destroys pulp life, and it is a close diagnosis to determine how much nature will tolerate in semi-electrocuting sensibility. There are all degrees of currents imposed upon nature in the practice of dentistry, from that mentioned in devitalizing pulps down to drinking ale from pewter mugs. When a gold crown or band extends beneath the gums, a current is established and the metal in touch with the tissue becomes an electrode. When a gold plug is inserted, it, too, is an electrode. In case of normal dentine it is insulated from the pulp and electricity is discharged from the surface where it received potential. When the dentine is a conductor, the current is abnormal to the functions of the pulp. The conductivity of dentine is governed by the proportion of moisture it contains. The principle in regard to currents is laid down in books as follows: " Feeble currents of electricity, continued for a long time, are equivalent to a stronger current for a shorter period." A developing tooth is a living organ, the pulp being its life centre. When for any external cause thermal changes produce pulp-disturbance, that is an indication that nature's process of calcification is being disturbed. In cases where teeth are worn nearly to the gums by slow degrees, secondary dentine fills the pulp-chamber and the pulp occupies only the root-canal. Invasion by caries is accompanied by acid which reverses nature's current. There is no building up against caries; thus decay in time reaches the pulp. Here we have another condition which produces secondary decay,—that is, caries around metal filling. When a metal plug enters sensitive dentine, cold—air, food, drinks, etc.—sends a shock to the pulp. I say shock because animal electricity heat ranges between freezing and 105°; thus in food and drinks the limits are nearly reached. We can readily see what the effect might be by introducing abnormal conditions so near a pulp. This one feature has been the hardest to understand on account of the difference between vital and physical electricity. Some might wish to know why a large gold plate would not cause more disturbance than a filling or gold crown. Because a plate rests upon gum tissue, which is a good conductor. The entire under surface being a conductor, there could be no concentration or discharge at any given point. Crowns that extend under the cervical border often effect the gums; so with fillings which extend into dentine sufficiently moist to be a conductor. Having thus far given most of our time to scientific teachings, I will illustrate science by practical application.

Remembering that Dr. J. T. Barker, who in a way is responsible for my presence this evening, more than a year ago showed much interest in clinics in my office, I will mention my practice in that line of work, which, as once remarked, is "filling made easy and more effectual." It relates to starting fillings of gold in shallow cavities located upon labial and buccal surfaces. I only venture to teach in this line because of much experience and the happy results from using crystal gold for a foundation, not claiming discovery of the principle. Many years ago I was equally interested, with Dr. Barker, in Dr. Howard's office in Buffalo, N. Y. If at the close of discussion some member should say, "I hope it will not go out that it is the sense of this meeting that it advocates the sticking in of gold fillings with varnish," it would not be the first time I have heard it. Call it what you will, the fillings stick. I have prepared a piece of ivory, which may be passed around, showing depth of cavities, with linings of varnish and of cement; also the crystal gold foundation, and again the first layer of gold-foil, and last a finished filling. There is no need of any one failing to do this work if the instructions are followed, and I have endeavored to explain so as to be understood; as above mentioned, success lies in the preparation of the gold foundation. Watt's crystal gold, No. 1, is the only preparation of gold that would enable me to do the work, which with it is easy. The drilling of pits or undercuts for starting fillings is unnecessary in this kind of cavity. In preparing the gold, cut from the cake, with a very *sharp, thin* blade, slices about seventeen plate gauge; the thickness of a small penny is near enough; when ready to use, cut the slice into pieces that will cover the cavity, anneal upon mica,—not over a flame, as the heat melts the fine crystals. Prepare the cavity according to decay. With good access and right-angle walls the thickness of twenty or twenty-two gauge is sufficient. No matter if deeper or shallower in places, or convex at bottom. For shallow cavities use varnish. Dry with hot air,—and that is of more importance than is generally understood. It is known that dentine is relieved of sensitiveness when desiccated, and while in that condition this varnish takes the place of moisture. A thin lining is thus formed which prevents the return of moisture, and the return of sensation also. On that account varnish is better than cement.

In introducing the filling, varnish and remove excess with small pieces of rubber dam; held in dressing-pliers, it takes up varnish and leaves no lint behind. While the varnish is tacky cover the cavity with a piece of gold, press the centre with a finely serrated, oval-faced plugger, working from the centre to margins, then with a thin flat serrated plugger, draw the overlapping portions towards the centre, and press back against the cavity wall into the corner, so as not to cut the gold. Pass around the margin and remove the excess of gold. If the gold is found broken or uneven, add another layer, not so large as the first. Upon that foundation build up the filling, using foil in tape form, made of foil folded in flat layers. Cement also makes a good foundation. Its use is indicated in deeper cavities. When the cavity has been prepared as above mentioned for varnish, use cement in like manner; make it quite thin, and spread it over the dentine as if it were heavy varnish. Place the gold over the cavity, and pack from the centre, forcing the cement out at the borders. Remove the cement and portions of gold that have been touched with it. As soon as the cement has set, so as not to be forced out by pressure, clean the enamel from any portions lodged there, and proceed to finish as described with varnish. In the use of amalgam for large cavities, particularly in devitalized teeth, cement linings are very important. It fills the porous dentine, also prevents chemical action upon the amalgam, as it always occurs when in contact with soft dentine. I should remark that, in filling deep cavities with cement to displace a portion of the amalgam, it is best to do the body of the filling with cement mixed for filling, and, when ready to finish with gold or amalgam, add a thin mixture, as would be used in shallow cavities, as gold or amalgam does not adhere to cement once set. This should be done in adding metals to old cement fillings.

Let us now consider the combination of gold and tin. There seems to be a wide difference of opinion regarding the use of tin at cervical borders for guard fillings. I will quote from the In-

TERNATIONAL DENTAL JOURNAL. In the October issue, 1898, may be found the discussion of Dr. Safford G. Perry's paper (page 567), "Treatment of Cervical Borders." One point of great importance was not settled to the enlightenment of readers. I will give my own conclusions and practice, which I believe will show that both the gentlemen were right from their stand-points. This subject has been made a study, and close observation has traced the causes to their respective conditions. There is no other metal used for filling teeth that is more compatible to dentine than tin. Tin and gold in the same cavity are in perfect harmony, nor is the tin dissolved. Tin- and gold-foil placed together in alternate layers make a durable filling resembling amalgam in color and hardness. Within a few years I saw several large fillings of this kind in molars which were done by the late Dr. Abbott, of Berlin. They had been done at the time over twenty years. This was noticeable: Upon the surfaces occasionally were visible specks of gold, also pits occasioned by the dissolving of tin. There was no decay around the plugs. These are facts which can be produced by observing the conditions. Again, I practised using tin at the cervical borders until I found beneath the gold at that point a black, soft mass. I do not remember seeing decay or sensitive dentine. I abandoned the use of tin for that purpose, and use amalgam when admissible. As Dr. Perry and Dr. Truman were earnest in the discussion, and Dr. Truman could not account for the difference, I trust they will kindly receive a suggestion. Quoting from Dr. Perry:

"Of course, you will understand that I am speaking of these three substances—gutta-percha, gold, and amalgam—only as foundations at the cervical border for oxyphosphate of zinc. I have not included tin, for while in some places I consider it the most perfect of all filling-materials, I do not trust it in sheltered places on proximate surfaces, and for the reason, of course, that it undergoes chemical dissolution. For this reason, many years ago, when it was advocated as well suited, pure or rolled with an alternate leaf of gold, for use along the cervical border, I distrusted it and used it but little. The few teeth that I ever filled in that way were afterwards repaired by replacing the softened tin by either gold or amalgam."

Dr. Truman remarked, "There were one or two points that arrested my attention during the reading of the paper that seemed to me to be of importance. When I heard Dr. Perry state that he could not use tin at the cervical border without it disintegrating, it seemed strange to me how we all differ on many points that are of practical importance. I remember that Dr. Jenkins, of Dresden, once said that he could not fill a cervical border with gold, that he had given that up as a practical impossibility, but that he could use tin and gold with perfect satisfaction. And I am forced to agree with him, not only as to the use of tin and gold, but tin alone when put in upon the cohesive principle, as I think all tin should be used. Of course, I bow always to Dr. Perry's large experience in this matter, and hesitate even to oppose that much of his paper."

The above shows different opinions upon a practical point without much benefit to the reader. As I view the matter, it is this: We know that gold and tin work well together when tin forms a considerable portion of the filling. Dr. Truman was right when he said, "Tin alone when put in upon the *cohesive* principle." The unseen fact is this: Gold by induction imparts to tin in contact a preserving property; that is, there is an interchange of atoms which forms an alloy of gold and tin which is insoluble. This alloy is only about the thickness of one layer of tin-foil. That, however, is enough to form an inseparable union between the metals. When the gold is put into an acid which will dissolve tin, it will be found that the surface retains the alloy unless a stronger agent is used to dissolve the alloy. When the tin is used cohesively, it represents a body of tin, the alloy cares for the joint and the tin is like a portion of a solid tin filling. On the other hand, the tin in case of two or three leaves does not enter into combination and it is subjected to galvanic action produced by the gold, and softening is the result. I would not risk gold and tin rolled together into a rope, but would feel safe with gold and tin placed together in alternate leaves and cut with scissors, provided the gold was placed against the dentine. One thing is certain, an *excess* of non-cohesive tin will unbalance the alloy and allow disintegration. Thus it may be seen that small, unseen causes produce varied results. While this point of induction is in mind, we will apply it to amalgam. Amalgam when added to an old plug forms a perfect union by the mercury entering into the old material. It so happens that still another patch may be needed. An observer will remember that in after years seams appear and the fillings seem to be separating; in fact, do separate in some cases, the cause being that the joint contains more mercury than other portions of the plug. The first portion absorbs mercury, which changes its proportion at the joint, and that thin strata becomes a positive element between the other elements more negative. To correct such effects, place a layer or two of gold-foil against the amalgam wall and introduce the filling. The gold disappears, but the influence remains, and the joint will be the last to give way, because the gold renders the joint negative. Lining cavities with tin under amalgam is good practice and next to cement. It presents an amalgam largely composed of tin, which, like tin, arrests caries and also blends the elements in the alloy which always exists in amalgam, that is, caused by the cuttings that are not fully amalgamated, with the other portions containing more mercury.

As this contribution will probably close my writing upon this special subject, it seems proper to remark that, in all the controversy that has been manifested for and against the points at issue, friendships have not been broken, and I accord to all who have opposed the principles advocated the same zeal and integrity in maintaining the dignity of the profession against what was believed to be debasing that I felt in promulgating what was believed to be science. On the other hand, I will ever remember the kind friends and agencies who have given aid and encouraged me through more than a score of years in which discussion has been going on. First, I will mention my friend Dr. Flagg, as the first cause, and the first to contribute a clinical foundation upon which to rest the "electro-chemical theory." His tabulated experiments, his clinical experience, his faithful and frequent correspondence, his teachings and liberal donations of the latest discovered materials, together with clinics in his laboratory as often as I could enjoy them, have been the main help in crystallizing theory into science. Second, the Dental Society of the State of New York, and the New York Odontological Society, from the first, have invited papers for discussion and have shown appreciation by high official appointments in my own State. Third, and to close, the Northeastern Dental Society has contributed the opportunity I now enjoy, of showing through its transactions to the dental world the relations of science to progress in dental practice. And science we define as a revelation from the Creator to man, as manifested in matter and recorded in natural laws, which are divine laws and from the same source as the laws revealed by inspiration, recorded in Holy Writ and accepted by faith. Therefore, by authority of natural laws and the testimony of clinical experience I maintain that the adaptation of filling-materials to the conditions of teeth stands upon a scientific basis.

